# Insecticidal activity of marigold *Tagetes patula* plants and foliar extracts against the hemipteran pests, *Lygus hesperus* and *Bemisia tabaci*

**DOI:** 10.1371/journal.pone.0233511

**Published:** 2020-05-19

**Authors:** Jeffrey A. Fabrick, Andrea J. Yool, Dale W. Spurgeon

**Affiliations:** 1 U.S. Department of Agriculture (USDA), Agricultural Research Service (ARS), U.S. Arid Land Agricultural Research Center, Maricopa, AZ, United States of America; 2 Adelaide Medical School, University of Adelaide, Adelaide, SA, Australia; Institut Sophia Agrobiotech, FRANCE

## Abstract

The western tarnished plant bug, *Lygus hesperus* Knight (Hemiptera: Miridae) and the whitefly, *Bemisia tabaci* Gennadius (Hemiptera: Aleyrodidae) are key hemipteran pests of numerous crop plants throughout the western United States and Mexico. Management in the U.S. currently relies on only a few insecticides and is threatened by the evolution of resistance. New chemistries or alternative management strategies are needed to reduce selection pressure on current insecticides and enhance control. Here, we investigated the bio-insecticidal toxicity of the French marigold, *Tagetes patula* Linnaeus (Asterales: Asteraceae), against both *L*. *hesperus* and *B*. *tabaci*. Assays indicated significantly reduced survival of both pest species on *T*. *patula* plants, and in diet incorporation assays containing aqueous and methanolic marigold foliar extracts. Mortality was concentration-dependent, indicating the presence of one or more extractable toxicants. These data suggest that *T*. *patula* plants have insecticidal constituents that might be identified and developed as novel alternatives to conventional chemical treatments.

## 1. Introduction

Many plants exhibit resistance to insect attack that typically arises from one or more mechanisms; tolerance, non-preference, or antibiosis [1–4]. Antibiosis (where plant defenses affect pest biology) is often facilitated by phytotoxins, some of which have been developed as botanical pesticides. Pyrethrum, nicotine, neem oil, essential oils, and rotenone are examples of phytochemicals used as commercial botanical pesticides [5–7]. Several more recently investigated plant extracts and essential oils also show promising activity as bio-insecticides [8–12] or repellents [13,14].

Several species of marigold (*Tagetes* spp.) are known to contain phytochemicals with pesticidal activity. For example, numerous studies have shown insecticidal activity associated with *Tagetes erecta* L. (African marigold), *T*. *minuta* L. (Mexican marigold), or *T*. *patula* L. (French marigold) against mosquitoes [15–20], sand flies [21], a leaf hopper [22], grain/seed beetles [23–26], termites [27], human head lice [28], bed bug [29], an aphid [30], and several caterpillars [31,32]. These marigold species have also shown activity as acaricides [33,34] and nematocides [35–37].

Numerous insecticidal compounds have been isolated from essential oils and root extracts of *Tagetes* spp. [30,38]. Monoterpenoids, carotenoids, and flavonoids are the major biocidal constituents of volatile oils from aerial plant parts of *Tagetes* spp. [30,38]. Photoactive thiophenes, present primarily in roots and flowers, are also biologically active against several insect species [19,23,39–41]. Unfortunately, many of these active compounds have limited practical use because they are volatile and have poor persistence under field conditions [5].

The western tarnished plant bug, *Lygus hesperus* Knight (Hemiptera: Miridae) and the whitefly, *Bemisia tabaci* Gennadius (Hemiptera: Aleyrodidae) are two major pests of cotton and other crops throughout the western United States [42–44]. Tarnished plant bug management typically relies on multiple applications of conventional insecticides of various chemical classes [45–46]. Whitefly control in the western U.S. is primarily dependent on insect growth regulators (IGRs) and neonicotinoids [46–47], although broad-spectrum insecticides are also used [46]. In Arizona, an Integrated Pest Management program has been implemented against both lygus and whitefly [44]. However, success of this program is dependent on the continued effectiveness of only a few insecticides [48]. Because these few chemicals have been widely used for many years and because both pest species have evolved resistance to numerous other insecticides [48–52], new control tactics are needed. Hence, the discovery of new compounds or complex mixtures of bioactive compounds with novel insecticidal modes of action are needed to reduce such selection pressure, and novel botanical insecticides could fill such pest management niches [9]. Here, we demonstrate that the marigold, *T*. *patula*, and its crude extracts, have toxicological activity against two important hemipteran pest species, revealing that future benefits might include use of foliar extracts for direct use as botanical insecticides as well as a resource for future isolation and development of beneficial compounds.

## 2. Materials and methods

### 2.1 Insects

Adult *L*. *hesperus* were obtained from a laboratory-reared colony maintained on common bean pods (*Phaseolus vulgaris* L.) and raw sunflower seeds (*Helianthus annuus* L.) as shown previously [53]. The colony was initiated from adults collected from alfalfa (*Medicago sativa* L.) on the University of Arizona Maricopa Agricultural Center farm, Maricopa, AZ. Because *L*. *hesperus* reared for more than three generations exhibit effects of laboratory selection [53], we examined only insects collected less than three generations from the field. Adults were collected from the colony within 24 h after adult eclosion.

Adult *B*. *tabaci* were from a Middle East-Asia Minor One (MEAM1) laboratory colony maintained at the U.S. Arid Land Agricultural Research Center, Maricopa, AZ. Whiteflies were continuously reared on *Brassica oleracea* L. within a 35 x 35 x 85-cm mesh cage in a greenhouse maintained between 21-32°C with ambient photoperiod [54]. To synchronize ages of whiteflies for bioassays, uninfested broccoli plants were placed into the cage harboring our whitefly lab colony. After 24-48 h the infested plants were removed from the cage and adult whiteflies were removed by shaking plants and/or gently brushing with paint brush. The plants with newly oviposited eggs were held in new cage within greenhouse for 1-2 weeks until initiation of adult whitefly emergence. Adult whiteflies were again removed by shaking/brushing. Then, newly emerged adult whiteflies were collected by aspiration within 24 h and used immediately in diet and on-plant tests.

### 2.2 Plant propagation

Seeds from the marigold, *T*. *patula* L. (Livingston Seed Co., Columbus, OH), were sown in pots containing filtered Sunshine Mix-1 (Sun Gro Horticulture, Agawam, MA) soil:sand mixture [9:5 soil:sand (v/v)]. Plants were grown in a greenhouse at 25 ± 8°C with 15-50% relative humidity (R.H.). Flowering marigold plants were harvested for leaf extractions or used for on-plant assays 3-5 months after seedling emergence. For on-plant assays, common bean (*P*. *vulgaris* L.) seed (Ferry-Morse, Norton, MA) and cotton (*Gossypium hirsutum* L.) seed (Bollgard II, Monsanto, St. Louis, MO) were sown and grown in the 9:5 soil:sand potting mix in a greenhouse using the conditions described above.

### 2.3 On-plant bioassays

**Fig 1:**
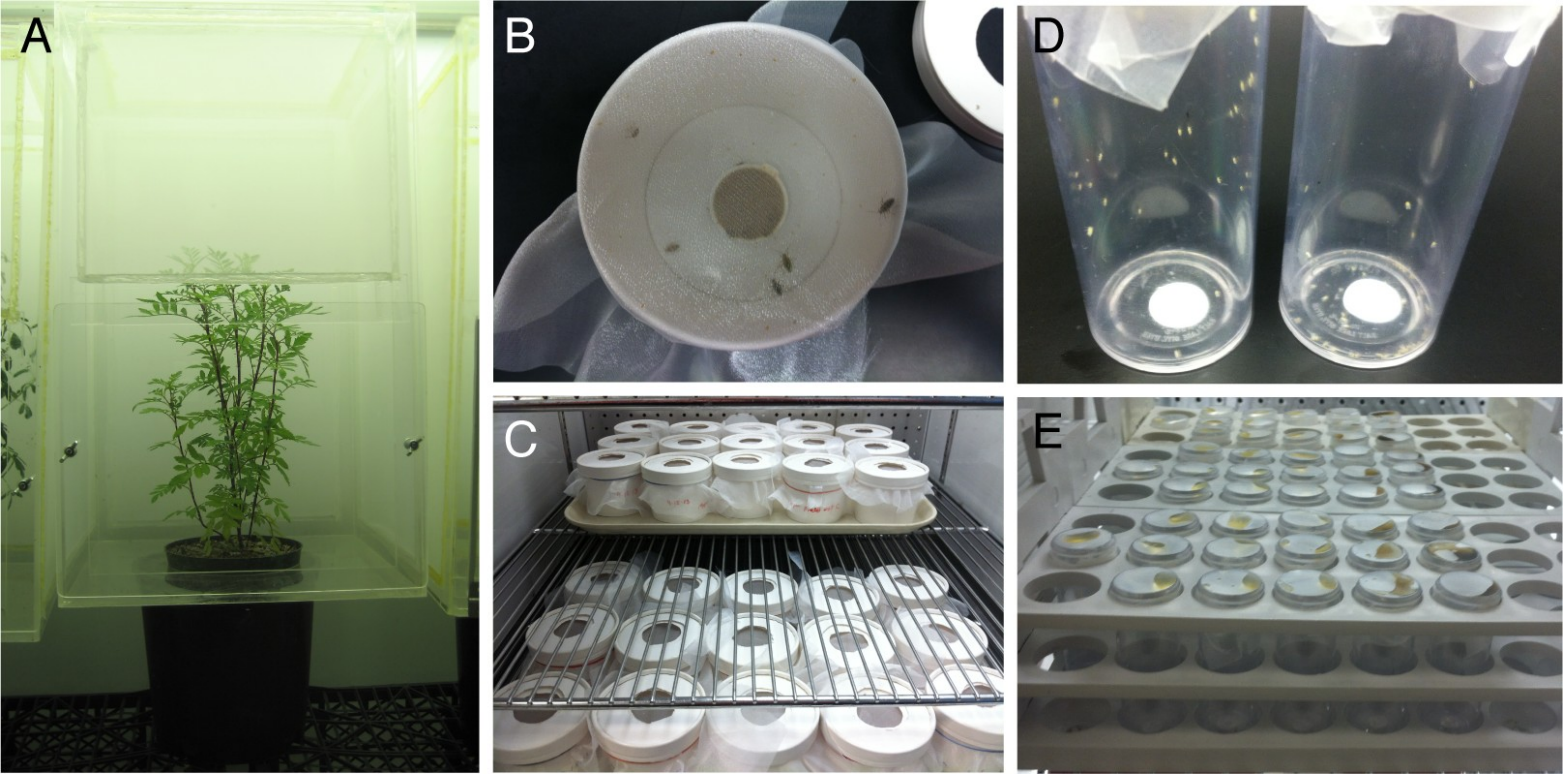
On-plant and diet-incorporation assays for adult *Lygus hesperus* and *Bemisia tabaci*. For on-plant assays (A), live adults were released into plexiglass cages with either marigold or control (common bean) plant and survival was assessed daily for 13 d. For incorporation assays, marigold extracts were mixed into diets for *L*. *hesperus* (B and C) or *B*. *tabaci* (D and E) and survival was recorded daily for up to 3 (*B*. *tabaci*) or 14 d (*L*. *hesperus*).

On-plant tests of *L*. *hesperus* survival were conducted in the greenhouse at 25 ± 8°C with a natural daylength of 10-12 h. Adults less than 24 h-old were collected, chilled on ice, and sorted into cohorts, each containing 20 males and 20 females. The 40 adults were simultaneously released into 37 x 37 x 61-cm plexiglass cages with mesh sides and a hole in the bottom that accepted a 3.8-liter pot (Fig 1A). Tap water was provided *ad libitum* in each cage via 30-mL vials sealed with foam plugs. Each of the three experimental repetitions included three each of *T*. *patula* test plants and three *P*. *vulgaris* control plants (six plants per repetition). Survival of the *L*. *hesperus* adults was recorded by counting the live and dead insects every 24 h for 13 d.

For *B*. *tabaci* on-plant assays, age-synchronized adults (<24-h old) of indeterminate sex were aspirated into 30-mL plastic vials and released into plexiglass cages (described above) containing test plants within a plant growth chamber (Conviron E8, Controlled Environments, Winnipeg, MB, Canada) at 26.0 ± 1.0°C, 40–60% R.H. under a 14:10 (L:D) h photoperiod. A total of 55-60 whiteflies were released into each cage. Survival of adults and numbers of eggs were counted every 24 h for 3 d. Each of the five experimental repetitions consisted of two each of the *T*. *patula* and two *G*. *hirsutum* plants per repetition.

### 2.4 Plant extractions

For aqueous extractions, 10 g of fresh marigold leaves were removed from mature plants, rinsed under tap water, cut into pieces, and placed into a mortar containing 30 mL of ultrapure water. Leaf material was crushed with a pestle for 5 min. After adding 20 mL of ultrapure water to rinse the pestle, the slurry was transferred to Erlenmeyer flask and total volume was brought to 150 mL with ultrapure water. The flask was covered with Parafilm M (Pachiney Plastic Packaging, Chicago, IL) and the contents were mixed with a magnetic stirrer for 2-3 hr. Equal amounts of the slurry were poured into each of four 50-mL capped centrifuge tubes and centrifuged at 10,000 x *g* for 10 min. Supernatants were vacuum filtered through Whatman #2 filter paper, then evaporated to dryness at 40°C on a heated stir plate. The dried residue was resuspended in 12 mL of ultrapure water and transferred to a pre-weighed centrifuge tube. Aqueous extracts were lyophilized using a benchtop lyophilizer (FreeZone 6 Liter, Labconco, Kansas City, MO). The dry extract was weighed and stored at 4°C until used in feeding bioassays.

For methanol extractions, 10 g of fresh marigold leaves were crushed with mortar and pestle in 30 mL of methanol (Fisher Scientific, Waltham, MA). The slurry was transferred to an Erlenmeyer flask, brought to 150 mL with methanol, covered, and stirred for 2-3 hr. The slurry was centrifuged and filtered as indicated above. Filtered supernatants were evaporated to dryness overnight at 30°C on a heated stir plate. The dried residue was resuspended in 12 mL methanol, transferred to a pre-weighed centrifuge tube, and dried in a fume hood under an air stream. Once dry, tubes were weighed and stored at 4°C.

### 2.5 Diet incorporation bioassays

To test marigold extracts for toxicity against *L*. *hesperus* and *B*. *tabaci*, we incorporated increasing concentrations of either aqueous or methanolic extracts into the food and observed mortality over time. For *L*. *hesperus*, aliquots of liquid diet [55] were mixed with dried soluble marigold extract [0 to 25000 parts per million (ppm)], or with methanol extract (0 to 10000 ppm) dissolved in 1% Tween 80 (Sigma, St. Louis, MO) (Fig 1B and 1C). Three-mL aliquots of diet mixtures were placed into inverted lids of 30-mL plastic vials and covered with tightly stretched Parafilm M. A brush was used to apply 15% (w/v) sucrose to the Parafilm to entice feeding by the insects. To account for possible buffer effects in the methanol extracts, 0 ppm doses contained the same volume of 1% Tween 80 used in extract samples. We repeated the assays three times, with each treatment replicated three times in each assay. Repetitions of the assays were conducted on different days and used fresh extract preparations each time. Lids containing the diets were each placed into the center of a 355-mL paper cup and 10 *L*. *hesperus* were released into each container (3 replicates x 5 doses x 3 repetitions x 10 adults per cup = 450 *L*. *hesperus* per test extract). Cups were covered with fine mesh secured with a rubber band. Feeding cups were held in a growth chamber at 26.0 ± 1.0°C, 40–60% R.H. under a 14:10 (L:D) h photoperiod. Survival and mortality was recorded by direct visualization and counting of live and dead insects every 24 h for up to 14 d.

For *B*. *tabaci* diet-incorporation bioassays, 50 adults (<24-h-old) were aspirated from *B*. *oleracea* into 30-mL plastic vials (Fig 1D), previously rubbed with a dye- and fragrance-free dryer sheet (Essential Everyday^TM^, SuperValu Inc., Eden Prairie, MN) to decrease static electricity. Each vial of insects was covered with stretched Parafilm M. Whitefly diet consisted of filter-sterilized 5% (w/v) Difco^TM^ yeast extract (Thermo Fisher Scientific, Waltham, MA) in 15% (w/v) sucrose with 1% Tween 80. Serial dilutions of marigold aqueous or methanol extracts (0 to 50,000 ppm) were incorporated into whitefly diet. Diet aliquots (0.3 mL) containing marigold extract were dispensed on top of the Parafilm stretched over each vial containing the test whiteflies (Fig 1E). Test vials were held in a growth chamber at 28.0 ± 1.0°C, 30% R.H. under a 14:10 (L:D) h photoperiod. Whitefly mortality was recorded at 0, 24, 48, and 72 h. Each repetition of the experiment consisted of three replicates for each dose of marigold extract (3 replicates x 5 doses x 3 repetitions x 50 adults per vial = 2250 *B*. *tabaci* per test extract).

### 2.6 Data analysis

For statistical analyses of on-plant survival assays and whitefly oviposition, we used Prism 7 for Mac OS X to plot and perform Analysis of Covariance (ANCOVA) to test whether the dose-response slopes obtained by linear regression differed among plant species. For diet-incorporation assays, survival analysis was performed using the LIFETEST procedure of SAS [56] and the Wilcoxon statistic. Multiplicity-adjusted comparisons among the survival functions of different doses within each extract were made using the ADJUST=SIMULATE option of the STRATA statement.

## 3. Results

### 3.1 Marigold toxicity to *Lygus hesperus*

**Fig 2:**
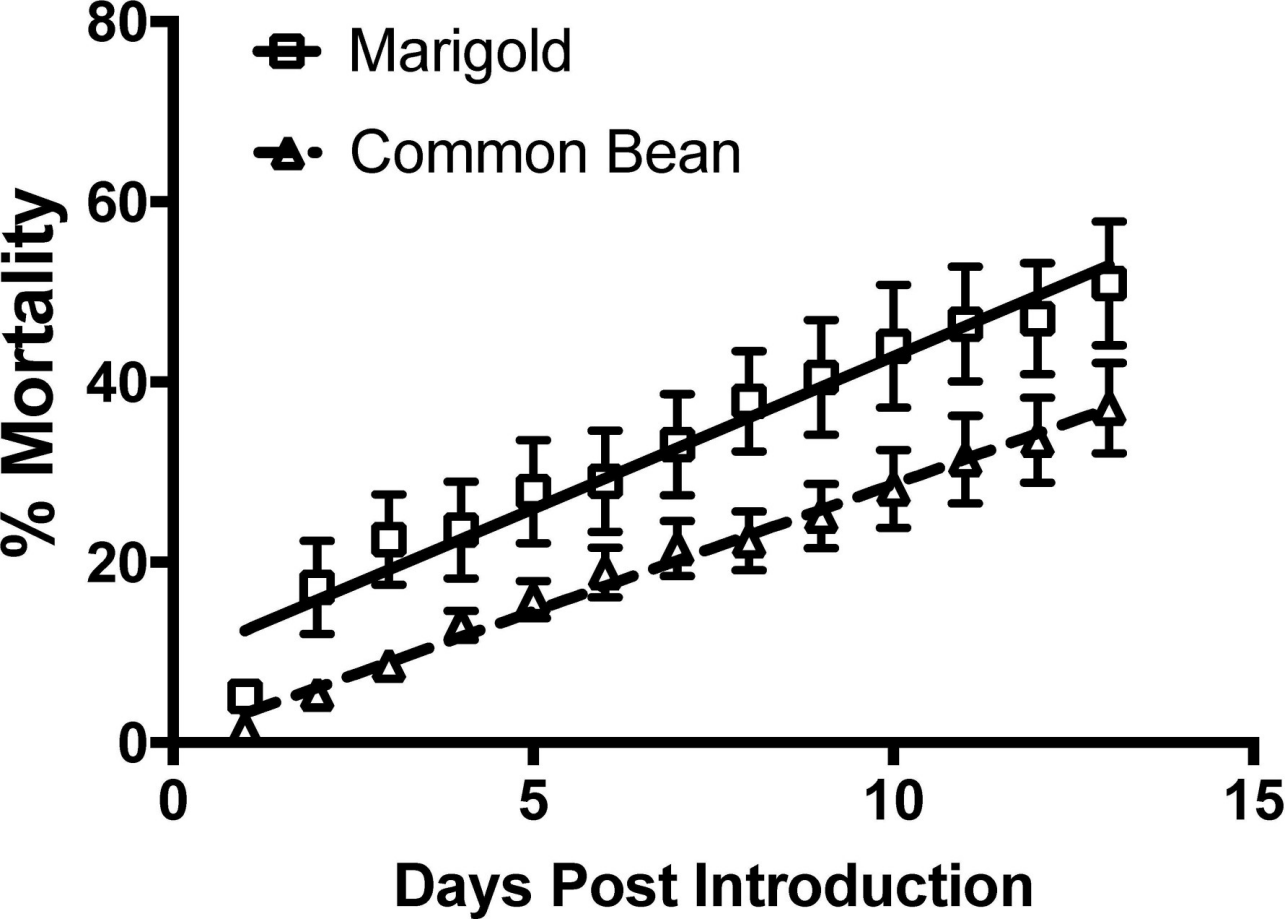
Mortality for adult *Lygus hesperus* on *Tagetes patula* L. (marigold) and *Phaseolus vulgaris* L. (common bean). Lines indicate the linear regressions and bars represent standard errors.

When *L*. *hesperus* adults were held on intact plants, mortality occurred more rapidly on marigolds compared with the common bean control (Fig 2; ANCOVA *F* = 6.23; df = 1,22; *P* = 0.021). The rate of mortality (%/d) was higher for those on marigold (slope ± SE, 3.38 ± 0.209; intercept ± SE, 9.05 ± 1.66; R² = 0.960; *F* = 261.6; df = 1,11; *P* < 0.001) than for those on common bean (slope ± SE, 2.82 ± 0.080; intercept ± SE, 0.448 ± 0.634; R² = 0.991; *F* = 1245; df = 1,11; *P* < 0.001).

### 3.2 Marigold toxicity and reduced oviposition in *Bemisia tabaci*

**Fig 3:**
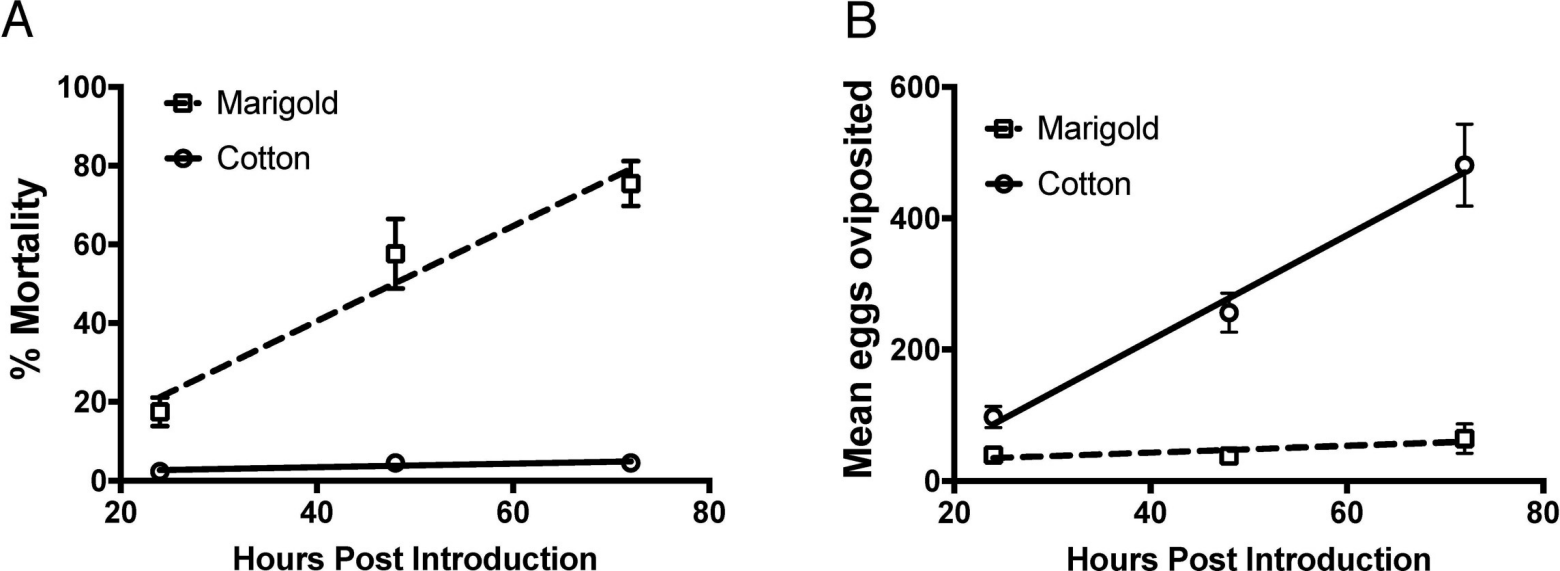
Mortality (A) and oviposition (B) of adult *Bemisia tabaci* on *Tagetes patula* L. (marigold) and *Gossypium hirsutum* L. (cotton). Lines indicate the linear regressions and bars represent standard errors.

The difference in *B*. *tabaci* on-plant mortality (%/h) between marigold and cotton plants was also pronounced (Fig 3A; *F* = 35.51; df = 1,56; *P* < 0.001). Mortality of *B*. *tabaci* on marigold occurred relatively rapidly (slope ± SE, 1.21 ± 0.193; intercept ± SE, -7.83 ± 9.99; R^2^ = 0.584; *F* = 39.30; df = 1,28; *P* < 0.001) whereas little mortality was observed on cotton (slope ± SE, 0.045 ± 0.031; intercept ± SE, 1.64 ± 1.59; R^2^ = 0.073; *F* = 2.20; df = 1,28; *P* = 0.149). Correspondingly, oviposition was reduced on marigold relative to cotton (Fig 3B; *F* = 34.24; df = 1,56; *P* < 0.001). On cotton, *B*. *tabaci* laid 8.00 ± 1.20 eggs per h (R² = 0.614; df = 1,28; *P* < 0.001), while on marigold the rate was 0.51 ± 0.44 per h (R² = 0.046; df = 1,28; *P* = 0.258).

### 3.3 Toxicity of marigold foliar extracts to *Lygus hesperus* and *Bemisia tabaci*

**Fig 4:**
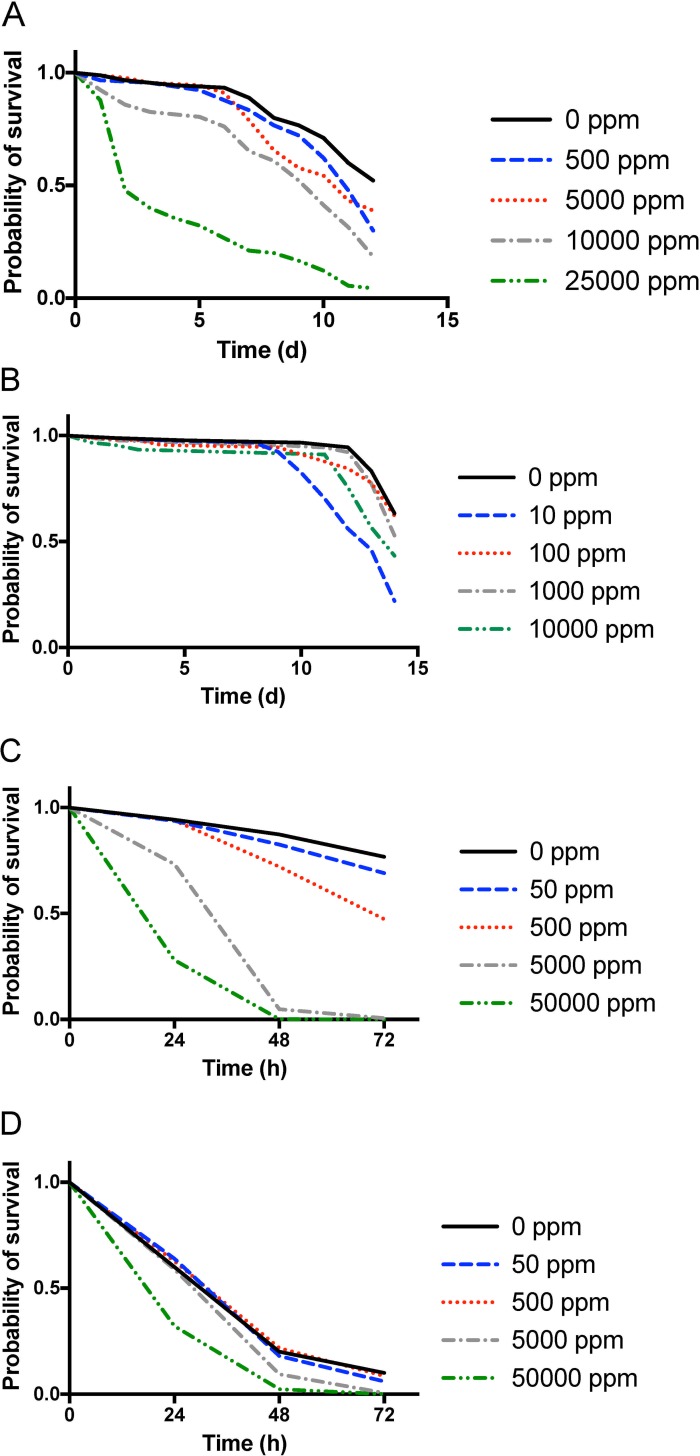
Survival functions of adult *Lygus hesperus* (A and B) and *Bemisia tabaci* (C and D) fed on *Tagetes patula* (marigold) foliar extracts incorporated into artificial diet. Aqueous (A and C) and methanol (B and D) extracts were fed for up to 14 d for *L*. *hesperus* and 72 h for *B*. *tabaci*, respectively.

Tests of aqueous foliar extracts from *T*. *patula* L. for toxicity against *L*. *hesperus* indicated a significant dose-mortality response (χ^2^ = 195.6, df = 4, *P* < 0.001; Fig 4A and Table 1). Although differences among the *L*. *hesperus* survival functions were indicated among doses of the methanol extracts (χ^2^ = 72.92, df = 4, *P* < 0.001), multiple comparisons showed only few doses that were significantly different (Fig 4B and Table 2). Furthermore, the mortality responses did not indicate a consistent dose-dependence. It was notable that the highest mortality was associated with the lowest concentration of the methanolic extract.

**Table 1:** Multiple comparisons among survival functions of *Lygus hesperus* adults fed diet containing doses of *Tagetes patula* (Marigold) aqueous extract.

Group comparison			
Dose (ppm)	Dose (ppm)	χ^2^	Adjusted-*P*^a^
0	500	1.3536	0.767
0	5000	0.9269	0.866
0	10000	13.8772	0.002
0	25000	124.1	<0.001
500	5000	0.0405	>0.999
500	10000	6.4505	0.081
500	25000	96.1438	<0.001
5000	10000	7.4896	0.049
5000	25000	99.5276	<0.001
10000	25000	49.7122	<0.001

^a^*P*-values from pair-wise comparisons were adjusted for multiplicity using the SIMULATE option of the LIFETEST procedure of SAS [56].

**Table 2:** Multiple comparisons among survival functions of *Lygus hesperus* adults fed diet containing doses of *Tagetes patula* (Marigold) methanol extract.

Group comparison			
Dose (ppm)	Dose (ppm)	χ^2^	Adjusted *P*^a^
0	10	48.7086	<0.001
0	100	0.3726	0.974
0	1000	1.6607	0.696
0	10000	10.6905	0.009
10	100	44.1560	<0.001
10	1000	32.7773	<0.001
10	10000	14.0683	0.001
100	1000	0.4945	0.957
100	10000	7.5419	0.046
1000	10000	3.8455	0.282

^a^*P*-values from pair-wise comparisons were adjusted for multiplicity using the SIMULATE option of the LIFETEST procedure of SAS [56].

Adult *B*. *tabaci* exhibited dose-dependent toxicity when fed both aqueous (χ^2^ = 5512, df = 4, *P* < 0.001; Fig 4C and Table 3) and methanolic marigold extracts (χ^2^ = 760.7, df = 4, *P* < 0.001; Fig 4D and Table 4), although differences among doses appeared less marked for the methanol extracts compared with the aqueous extracts.

**Table 3:** Multiple comparisons among survival functions of *Bemisia tabaci* adults fed diet containing doses of *Tagetes patula* (Marigold) aqueous extract.

Group comparison			
Dose (ppm)	Dose (ppm)	χ^2^	Adjusted *P*^a^
0	50	6.7599	0.071
0	500	93.9796	<0.001
0	5000	1670.8	<0.001
0	50000	3189.1	<0.001
50	500	49.4215	<0.001
50	5000	1438.1	<0.001
50	50000	2836.3	<0.001
500	5000	952.9	<0.001
500	50000	2099.3	<0.001
5000	50000	171.9	<0.001

^a^*P*-values from pair-wise comparisons were adjusted for multiplicity using the SIMULATE option of the LIFETEST procedure of SAS [56].

**Table 4:** Multiple comparisons among survival functions of *Bemisia tabaci* adults fed diet containing doses of *Tagetes patula* (Marigold) methanol extract.

Group comparison			
Dose (ppm)	Dose (ppm)	χ^2^	Adjusted *P*^a^
0	50	3.4724	0.338
0	500	9.8047	0.015
0	5000	18.8668	<0.001
0	50000	421.3	<0.001
50	500	1.5773	0.720
50	5000	37.6432	<0.001
50	50000	488.5	<0.001
500	5000	54.5591	<0.001
500	50000	545.0	<0.001
5000	50000	255.6	<0.001

^a^*P*-values from pair-wise comparisons were adjusted for multiplicity using the SIMULATE option of the LIFETEST procedure of SAS [56].

## 4. Discussion

Concerns for the impact of pesticides on human health and the environment, as well as the constant evolution of pesticide resistance, necessitates a renewed interest in the discovery and use of novel non-synthetic bio-pesticides [6,57,58]. Many plants, including those from the Asteraceae family to which *Tagetes* spp. belong, contain antibiosis, antifeedant, or repellant compounds [5–6] with potential as pest management resources. Due to the substantial delay between discovery and commercialization of new bio-pesticides, only a few of the many promising plant-based compounds have come to market [5,7]. The dearth of commercialized botanical insecticides is due to regulatory limitations in the approval of products, inconsistency in the performance or delivery of active compounds, and limited availability of resources to pursue commercialization [5]. Limitations in resources often involve barriers to economical production or formulation of the active compounds. Here, our approach was to test both *T*. *patula* L. whole plants and minimally-processed leaf extracts for inherent activity against two arthropod pests endemic to the western U.S. and elsewhere. Our findings suggest that marigolds are a relatively untapped resource for the future identification, development, and commercialization of bioactive pesticidal compounds and/or for implementation of minimally-processed, whole plant-based insecticidal materials that might by-pass some of the delays in commercial pesticide development.

Because whiteflies and mirid bugs are global pests of many important crops, there is interest in using repellent plants in companion plantings [59–60]. Examples exist that demonstrate potential benefits of repellent companion crops for management of whiteflies [61,62], and efficacy of marigolds as companion plants has been reported for several insect pests [63–68]. In the southwestern U.S., while cotton is a suitable host, both pests prefer other plants [69–70], which lends itself to the use of cultural control strategies. For example, alfalfa (*Medicago sativa* L.) planted adjacent to cotton can act as a “trap crop” to reduce lygus damage [71–73]. Cotton can also be intercropped with plants that repel or deter *L*. *hesperus* and *B*. *tabaci* [59–60]. Studies show the potential management benefits of such companion plants for these pests at a relatively small scale [61,62]. However, the biotic mechanisms that underlie such repellency are often not well understood [74] and the use of this management tactic requires establishment and maintenance of the repellent plants. Furthermore, although Conboy et al. (2019) [62] showed that *T*. *patula* L. can serve as a repellent companion plant against the whitefly *Trialeurodes vaporariorum* Westwood on tomatoes in greenhouses, it is unknown what, if any, benefits would be achieved in lesser-value crops and/or in open field settings. Hence, additional research is needed to assess marigolds as companion plants for their effectiveness, feasibility, and economics for different crops under real-world conditions. Alternatively, extracts of bioactive compounds from plants such as *T*. *patula* may be developed as novel insecticides [75,76].

Our results indicate that *T*. *patula* possesses intrinsic insecticidal activity against two important hemipteran pests, *L*. *hesperus* and *B*. *tabaci*. We observed significantly reduced oviposition by *B*. *tabaci* on *T*. *patula* plants and significantly higher mortality for both *L*. *hesperus* and *B*. *tabaci* adults on marigold compared with control plants. These results could reflect altered behavior (e.g., repellency or feeding deterrence) rather than direct intoxication. However, we also observed dose-dependent mortality in diet-incorporation bioassays for both *L*. *hesperus* and *B*. *tabaci* adults. Differences between the dose-dependency of aqueous extracts, compared with methanolic extracts, may also provide useful insights. The clear dose-dependency of mortality associated with the aqueous extracts suggest minimal if any feeding deterrence. In contrast, dose-dependency (or inverse dose-dependency for *L*. *hesperus*) observed for the methanolic extracts suggest a methanol-soluble constituent (likely the toxicant) may have deterred feeding by the insects.

Although it was difficult to ascertain whether all insects within the replicates fed equivalently on the diet, several lines of evidence suggest the effect of the marigold extracts was manifested through toxicity rather than starvation. Feeding by both *L*. *hesperus* and *B*. *tabaci* on artificial diets containing marigold extracts was qualitatively indicated by 1) the aggregation of adult insects on provided diet, 2) direct observation of insects probing the diet through the Parafilm, 3) conspicuous reductions in the volumes of artificial diet with increased time of exposure, and 4) presence of dark green or brown excrement (the color of incorporated marigold extracts) within the assay containers (J.A.F., personal observation). Because processing of the aqueous and methanol extracts involved several drying steps at elevated temperature, most of the volatile organic compounds were likely removed and therefore not responsible for the responses we observed. Hence, we suggest that the foliage of *T*. *patula* likely contain compounds that are toxic against both *L*. *hesperus* and *B*. *tabaci* adults which are not innately repellant. Furthermore, our preliminary results indicate a direct impact on *L*. *hesperus* and *B*. *tabaci*, as the marigold extracts appeared to alter water transport and/or osmotic balance by targeting insect aquaporin proteins when expressed in a heterologous system (J.A.F. and A.J.Y., unpublished data).

Our results indicate *T*. *patula* possesses insecticidal constituents that may be useful as novel insecticides against mirid or whitefly pests. Before commercial implementation, residual traces of marigold phytochemicals would need to be evaluated for potential off-target effects against natural enemy and other beneficial arthropod communities [77] as well as unintended health impacts in human consumers. However, marigold extracts have been used for medicinal and dietary purposes by humans since ancient times. As reviewed by Gupta and Vasudeva (2012) [78], species of *Tagetes* have served as traditional medicinal herbs in Mexico and India for treating a variety of ailments. Aqueous and alcoholic extracts of the leaves have been used for example as antimalarials, anti-inflammatory agents, diuretics, treatments for gastrointestinal disorders in humans, and nematicides and insecticides in pests. Hence, further investigation is needed to identify specific toxicants and their modes of action in order to assess their potential value as alternatives to conventional pesticides.
